# Psychometric Properties and Measurement Invariance of the Cognitive Emotion Regulation Questionnaire in Chinese Adolescents With and Without Major Depressive Disorder: A Horizontal and Longitudinal Perspective

**DOI:** 10.3389/fpsyt.2021.736887

**Published:** 2021-10-22

**Authors:** Fengjiao Ding, Xin Wang, Chang Cheng, Jiayue He, Haofei Zhao, Daxing Wu, Shuqiao Yao

**Affiliations:** ^1^Medical Psychological Center, The Second Xiangya Hospital of Central South University, Changsha, China; ^2^Medical Psychological Institute of Central South University, Changsha, China; ^3^China National Clinical Research Center on Mental Disorders (Xiangya), Changsha, China; ^4^Affiliated Wuhan Mental Health Center, Tongji Medical College of Huazhong University of Science and Technology, Wuhan, China

**Keywords:** CERQ, measurement invariance (MI), emotion regulation, depression, Chinese high school students

## Abstract

**Objective:** The purpose of this study was to examine the psychometric properties and posited nine-factor structure of the Chinese version of the Cognitive Emotion Regulation Questionnaire (CERQ-C) in high school students and adolescents with major depressive disorder (MDD), including assessment of measurement invariance of CERQ-C and its subscales across gender, time, and presence of depression.

**Methods:** Chinese high school students from Hunan Province (*N* = 1,253) and adolescents with major depressive disorder (MDD) from the Medical Psychological Institute outpatient clinic at The Second Xiangya Hospital (*N* = 205) were enrolled. We examined the reliability, and model fit of the CERQ-C. Multigroup confirmatory factor analysis (CFA) was used to test measurement invariance of the subscales across gender, time, and presence of depression.

**Results:** The CERQ-C subscales showed good internal consistency and moderate *test-retest* reliability in high school students and excellent internal consistency in adolescents with MDD group. The nine-factor model yielded adequate fit indices in different samples. Multigroup CFA confirmed that CERQ-C is strongly equivalent across gender, time, and presence of depression.

**Conclusions:** The CERQ-C is a valid, reliable, and stable instrument for the evaluation of the cognitive emotion regulation (ER) strategies for different samples, including high school students and adolescents with MDD. The horizontal and longitudinal equivalences are strongly established.

## Introduction

Emotion regulation (ER) strategies are recognized as being important for overcoming chronic and acute adversity and sustaining one's well-being. Gross (1) defines ER as, “the process by which individuals influence which emotions they have, when they have them, and how they experience these emotions.” Maladaptive ER strategies have been reported to mediate the impact of adverse life events on mental health (2, 3). In a survey study of 206 adolescents focused on ER and aggressive behavior, Silk et al. (4) found that adolescents who reported highly intense and unstable emotions and relatively ineffective ER tended to have heightened levels of depressive symptoms and problematic behavior.

Cognitive ER strategies are seen as an effective way of coping with the processing of emotionally arousing information (5). Garnefski et al. (6) developed the CERQ to measure cognitive ER strategy use. The CERQ contains nine subscales, each of which targets a mood modulating strategy that individuals used after a negative experience: Self-blame, Rumination, Catastrophizing, Blaming others, Acceptance, Positive refocus, Refocus on planning, Positive reappraisal, and Putting into perspective. The first four are considered maladaptive, and have been found to be directly associated with being poorly adjusted, depression and anxiety. The remaining five strategies are considered to be adaptive because they are thought to be protective factors under stress. The use of different cognitive ER strategies has been shown to lead to different emotional experiences and behaviors. For example, Self-blame and Rumination are associated with depression, anxiety, and other psychological disorders (7, 8), and may interfere with one's ability to solve problems (9). Meanwhile, Positive reappraisal and Refocus on planning have been identified as protective factors (10). The CERQ is a widely used psychometric tool and has been translated into many languages and validated in diverse samples, including French (11), Chinese (12), Turkish (13), Persian (14), Spanish (15), and Arabic (17) samples, among others. Notably, adequate reliability and validity have been demonstrated in adolescent samples (11, 16). The Chinese-language CERQ (CERQ-C) has been shown to perform excellently in adults and in specific patient populations (12, 18), but has not yet been validated for Chinese adolescents. The original nine-factor structure of the CERQ has mostly been affirmed across samples (12, 16, 18, 19), however, a five-factor structure was supported in a sample of adults in remission from recurrent depression (20).

Adolescents undergo a rapid transformation in emotion response. They experience frequent, intensely emotional conflict and face more serious emotional difficulties than preadolescent children and adults. CERQ is widely used to assess the adolescents' cognitive ER strategies in researches. In a study in a sample of 248 Spanish adolescents examined the relationships between the use of cognitive emotion regulation strategies, physical-verbal aggression, and depression and found cognitive emotion regulation strategies related to physical-verbal aggression and depressive symptoms (21). In a study of Chinese adolescents, Chen et al. (22) found that the use of more active cognitive coping, both adaptive and maladaptive, was associated with less unprotected sex. Another study surveyed 1,265 Chinese adolescents and found cognitive ER strategies have partial multilevel mediation effects on the link between neuroticism and depressive symptoms (23).

Because stressful events are frequently encountered, an adolescent's ability to cope with them and to regulate their emotions plays an important role in the relationship between stress and psychopathology (24). A stable and excellent cognitive ER strategies instrument is necessary for adolescent to assess their strategies. Theoretically, the CERQ could provide a useful metric with which to elucidate emotionality and cognitive ER strategies use in adolescents, it logically follows that the examination of measurement equivalence across diverse groups is one necessary pre-requisite for CERQ to examine one's cognitive ER strategies in diverse contexts. Measurement invariance involves examining the psychometric properties of a scale across time (longitudinal measurement invariance) and/or across groups (25). Measurement invariance for many language versions of the CERQ has been supported across sociodemographic groups (11–13, 26, 27). But the measurement equivalence, especially the longitudinal equivalence and measurement invariance across the presence of depression, in Chinese adolescents has never explored before. Because it has been supposed that cognitive ER strategies may be related to major depressive disorder (MDD) (28), there is an interest in establishing measurement invariance across the presence and absence of depression. Regarding the use of cognitive ER strategies, some have found that girls tend to use more Self-blame and Catastrophizing than boys (29), while some have found no gender differences [e.g., (30)]. The measurement equivalence across gender is necessary. (31) found that early adolescents use cognitive ER strategies less frequently than late adolescents or adults, and that late adolescents score lower than adults on five subscales of the Cognitive Emotion Regulation Questionnaire (CERQ): Acceptance, Rumination, Refocus on planning, Positive reappraisal, and Putting into perspective. A pile of studies also found that as adolescents grown up, the use of cognitive ER strategies changed (32, 33). Moreover, many studies have utilized the CERQ to conduct follow-up researches and to assess the effect of intervention and treatment (34, 35). The longitudinal comparison of cognitive ER strategies made the establish of measurement equivalence cross time in adolescent population significance and value.

The main objective of the present study is to examine the measurement invariance of the CERQ-C among Chinese adolescents. First, we will examine the reliability and validity of the full scale and of its subscales; the factor structure will be analyzed using confirmatory factor analysis (CFA). Then, we'll evaluate the measurement invariance of the CERQ-C across gender, time, and presence of depression.

## Method

### Participants

This research was approved by the Ethics committee of Second Xiangya Hospital, Central South University. Using posters and advertisements, the high school student sample was recruited from two high schools in Hunan Province. The group-administered paper-pencil measure was taken to collect data from volunteered participants with two well-trained psychological post-graduate researchers' guidance in a quiet room. Structured clinical interview using Kiddie Schedule for Affective Disorders and Schizophrenia was conducted by two psychiatrists with students who scored high on the depressive scales, histories of any mental disorder were not eligible for participation. A clinical sample consisting of adolescent patients diagnosed with MDD were recruited at the outpatient clinic of Second Xiangya Hospital by two psychiatrists. Participants of adolescents with MDD were assessed with Kiddie Schedule for Affective Disorders and Schizophrenia, and findings reflective of any mental disorder besides MDD were exclusionary. The demographic characteristics of the high school student and adolescents with MDD samples are shown in Table 1. Notably, in both samples, the participants ranged from 15 to 18 years old. The male and female groups in the high school student sample were of similar ages, with males having a mean age of 16.28 (±1.038) years and females having a mean age of 16.43 (±0.965) years.

**Table 1:** Demographic characteristics of participants.

**Characteristic**	**High school students** **(***N*** =1,253)**	**Adolescents with MDD** **(***N*** = 205)**	**Statistics**	***P*** **(2-tailed)**
Gender, no. males (%)	629 (50.2)	57 (27.8)	χ^2^ = 34.243	<0.001
Mean age (SD)	16.36 (1.004)	16.43 (1.572)	*t* = 13.48	<0.001
Ethnicity, *N* (%)			–	–
Han	1,253 (100)	205 (100)		
Other	0	0		
Grade, *N* (%)			χ^2^ = 19.277	<0.001
10th	356 (28.5)	88 (42.9)		
11th	412 (32.8)	62 (30.2)		
12th	485 (38.7)	55 (26.9)		
Region, *n* (%)			χ^2^ = 26.208	<0.001
City	518 (41.3)	124 (60.5)		
Urban	735 (58.7)	81 (39.5)		
**Psychometrics, mean score (SD)**				
SAI	44.40 (10.07)	54.03 (8.22)	*t* = −14.576	<0.001
TAI	46.34 (9.33)	55.82 (7.84)	*t* = −2.878	0.004
CES-D	37.51 (8.92)	52.65 (8.21)	*t* = −22.715	<0.001
CD-RISC	57.82 (14.41)	44.16 (11.37)	*t* = 15.269	<0.001

*MDD, major depressive disorder; SAI/TAI, State/Trait Anxiety Inventory; CES-D, Center for Epidemiologic Studies Depression Scale; CD-RISC, Connor-Davidson Resilience Scale*.

### Procedure

Prior to the initial assessment, letters of informed consent were given to prospective participants and their parents detailing the aims of the study. All participants provided written informed consent before completing the study questionnaires. During the initial assessment, they completed a demographic form and the four psychometric scales (described below). Participants who did not complete all the questionnaires (*N* = 28) were excluded. To examine test-retest reliability and measurement invariance of the CERQ-C, 1 month following the initial assessment, a CERQ-C retest was administered to a subset of 594 high school participants.

### Instruments

#### Cognitive Emotion Regulation Questionnaire (CERQ)

The 36-item CERQ-C was used to evaluate cognitive ER strategies adopted by individuals when they are under pressure or facing negative events. It contains the following nine conceptually distinct subscales, each consisting of four items: Self-blame, Acceptance, Rumination, Positive refocus, Refocus on planning, Positive reappraisal, Putting into perspective, Catastrophizing, and Blaming others. Each item response was measured on a five-point Likert scale ranging from 1 (almost never) to 5 (almost always). Higher scores on a subscale indicated more use of that strategy. The CERQ-C showed good to very good internal consistency for all subscales (12).

#### Connor-Davidson Resilience Scale (CD-RISC)

The CD-RISC was used to evaluate individuals' resilience when facing adversity (36). The items were graded on a 5-point Likert scale from 0 (not true at all) to 4 (true nearly all the time). Higher scores represented better resilience. The 25-item Chinese community version of CD-RISC showed good reliability (37).

#### Center for Epidemiologic Studies Depression Scale (CES-D)

Levels of depression were evaluated with the CES-D (38), which measures the frequency of depressive symptoms experienced in the last week. The CES-D contains 20 items, each rated on a 4-point Likert scale from 1 (rarely or never, <1 day/week) to 4 (all the time, 5–7 days/week). Higher scores indicated a higher level of depression. The Chinese version of the CES-D has high reliability (39).

#### State-Trait Anxiety Inventory (STAI)

The STAI was administered to estimate levels of anxiety. The STAI is a 40-item self-report inventory that contains two 20-item subscales: state anxiety and trait anxiety (40). The state anxiety inventory (SAI) is designed to measure the respondent's characteristic feeling at the moment, while the trait anxiety inventory (TAI) is designed to measure the respondent's permanent and steady characteristic. All items were answered on a four-point Likert scale, with a higher total score indicating greater anxiety. The Chinese version of STAI has been shown to have satisfactory reliability and validity (41).

### Statistical Analysis

The survey data were analyzed in SPSS (v. 22.0). First, descriptive analyses were conducted to establish distributions of the data. Then the data were evaluated for reliability. The internal reliability of the scale was examined using Cronbach's α values, Cronbach's α-value >0.60 was considered acceptable (42, 43). The test-retest reliability was assessed by the intraclass correlation coefficient (ICC). ICC values of 0.00–0.20 denote slight reliability, 0.21–0.40 fair, 0.41–0.60 moderate, 0.61–0.80 substantial, and ≥0.81 almost perfect reliability (44). Based on the above classifications, ICC values of >0.60 were considered to demonstrate a high consistency of scale ratings. Considering the strong connection between the cognitive ER strategies and depression mentioned above, the criterion-related validity in each studied group was evaluated by an analysis of correlation between the CERQ subscales and BDI, S-TAI. Then two hierarchical multiple regression analyses (MRAs) were performed with depression as the dependent variable, with controlling for symptoms of anxiety (scores of SAI) (method = enter), nine cognitive emotion regulation strategies were the independent variables. Likewise, two hierarchical MRAs were performed with regard to anxiety (scores of SAI) as dependent variable, with controlling for depression (method = enter), nine cognitive emotion regulation strategies were the independent variables.

The factor structure of the CERQ-C was obtained via confirmatory factor analysis (CFA) to verify whether the nine-factor CERQ was a good fit for Chinese adolescents. CFA was conducted using M-plus 7.4 software (45). In order to allow more sophisticated statistical analyses, we treated the score as interval level data (a scale which Likert-type items possess 5 or more levels could be treated as interval), the analysis employed the maximum likelihood (ML) estimator (46, 47). Because of our relatively large sample size and the low sensitivity of the chi-squared test, we evaluated model fit with the comparative fit index (CFI), Tucker-Lewis index (TLI), and root mean square error of approximation (RMSEA). CFI ≥0.90, TLI ≥0.90, and RMSEA ≤ 0.08 were considered acceptable (48). To ensure that adolescents with MDD and gender group differences in CERQ-C subscale scores were not due to measurement artifacts; multi-group CFAs was used to examine measurement invariance across samples (39, 49).

After identification of the fitting model, four procedures were conducted to assess CERQ-C measurement invariance across presence of depression, the healthy group (non-depressive, high school students) was high school students, the depressive group was adolescents with MDD. First, configural invariance was examined to confirm factor equivalence between groups. Configural invariance means that the number of factors and pattern of loadings is the same for both groups. In the analysis of configural equivalence, the intercepts of the observed variables and residuals, and all parameters, were freely estimated. Second, metric invariance was then assessed to examine the equivalence of factor loading across groups. Metric invariance means that not only are the same items loading on the same factors for both groups, but the actual magnitude of the loadings is the same across groups for each respective Item. In the metric invariance analysis, the factor loadings were constrained to be equivalent for the groups to determine whether items represented the same concepts in both groups. Third, scalar invariance was evaluated with the constraint of item thresholds to equality across groups, equality of item thresholds indicates that the mean levels of the latent constructs are equivalent between groups. Fourth, strict invariance was evaluated to determine whether error variance was equivalent across groups. Each procedure was executed on the condition that the preceding condition was satisfied. Then measurement invariance tests were conducted across gender [samples were from high school students (1,253): group 1 was 629 males, group 2 was 624 females] and time [samples were from high school students, first time was high school students (1,253), second time was 1-month retest for 594 high school students], through the same processes. The measurement invariance tolerance levels were: ΔCFI <0.010, ΔTLI <0.010, and ΔRMSEA <0.015 (50).

After examining measurement invariance, we compared the high school student and adolescents with MDD groups, and we compared the males and female subgroups within the high school student group. *P* < 0.05 were suggestive of a significant difference. Standardized inter-group differences were established based on effect size (Cohen's d), such that 0.2< Cohen's d <0.5 indicated a small effect, 0.5< Cohen's d < 0.8 indicated a medium effect, and Cohen's d >0.8 indicated a large effect (51). Lastly, we performed Pearson correlations between the CERQ-C subscales and other scales, including the CES-D, STAI, and CD-RISC.

## Results

### Descriptive Statistics

The characteristics of high school student sample and adolescents with MDD sample are summarized in Table 1. Notably, these two groups differed in measures of anxiety, depression, and resilience.

### Reliability

The full CERQ-C and all CERQ-C subscales were found to have adequate internal consistency relative to an acceptability threshold of Cronbach's α = 0.7. The Cronbach's α (range, 0.777–0.900), mean inter-item correlation (range, 0.469–0.681), and 1-month test-retest reliability (ICC) (range, 0.507–0.717) values of the high school student group are reported in Table 2. Among the subscales, the Refocus on planning subscale had the highest Cronbach's α as well as the highest test-retest reliability statistics. Meanwhile, the Self-blame and Positive refocus subscales had the lowest Cronbach's α and test-retest reliability statistics, respectively. The ICC for the CERQ-C total scale was 0.677, which showed an acceptable test-retest reliability. The adolescents with MDD group obtained excellent Cronbach's α (range, 0.848–0.911), mean inter-item correlation (range, 0.585–0.720), Rumination had the highest Cronbach's α value.

**Table 2:** Reliability of the CERQ-C total scale and subscales.

**Scale**	**High school students**				**Adolescents with MDD**	
	**Cronbach's alpha**	**Mean inter-item correlation**	**ICC**	**95%CI**	**Cronbach's alpha**	**Mean inter-item correlation**
Self-blame	0.777	0.469	0.529	0.432–0.609	0.857	0.600
Acceptance	0.841	0.570	0.543	0.467–0.608	0.862	0.611
Rumination	0.870	0.627	0.534	0.472–0.590	0.911	0.720
Positive refocus	0.848	0.582	0.507	0.442–0.566	0.848	0.585
Positive reappraisal	0.894	0.681	0.656	0.602–0.704	0.908	0.714
Refocus on planning	0.900	0.691	0.717	0.676–0.754	0.868	0.624
Putting into perspective	0.844	0.575	0.587	0.532–0.638	0.855	0.600
Catastrophizing	0.890	0.672	0.564	0.507–0.616	0.908	0.713
Blaming others	0.846	0.583	0.517	0.455–0.574	0.846	0.585
Total scale	0.883	0.173	0.677	0.594–0.741	0.901	0.202

*CERQ-C, Chinese version of the Cognitive Emotion Regulation Questionnaire; MDD, major depressive disorder*.

### Convergent Validity

The Pearson correlations showed that the highest Pearson correlations between CERQ subscales, and symptoms of depression and anxiety were catastrophizing in both groups (Table 3). In adolescents with MDD group, the correlations between Self-blame, Rumination, and depression were also meaningful. In both healthy group and depressive group, resilience (CD-RISC scores) had an acceptable positively bivariate Pearson correlations with Positive refocus, and Positive reappraisal CERQ-C subscores, which are all considered adaptive coping strategies, while in high school student sample, resilience had highest negatively the highest bivariate Pearson correlations with Catastrophizing which represent maladaptive coping strategies (Table 3).

**Table 3:** CERQ-C subscale score correlations with the depression, anxiety, and resilience.

**Sample scale**	**Self-blame**	**Acceptance**	**Rumination**	**Positive refocus**	**Positive reappraisal**	**Refocus on planning**	**Putting into perspective**	**Catastrophizing**	**Blaming others**
**High school students**									
SAI	0.184**	−0.070*	0.224**	0.042	0.029	−0.169**	0.170**	0.396**	0.167**
TAI	0.241**	−0.066	0.256**	0.032	−0.052	−0.281**	0.166**	0.503**	0.205**
CES-D	0.230**	0.007	0.288**	0.090**	−0.003	−0.174**	0.203**	0.471**	0.253**
CD-RISC	−0.005	0.177**	−0.028	0.137**	0.414**	0.498**	−0.021	−0.303**	−0.112**
**Adolescents with MDD**									
SAI	0.062	0.027	0.279**	−0.067	0.024	−0.176*	0.029	0.392**	0.217**
TAI	0.209**	0.072	0.258**	−0.017	0.086	−0.203**	0.032	0.422**	0.148*
CES-D	0.344**	0.226**	0.361**	0.015	0.171*	0.006	0.087	0.499**	0.212**
CD-RISC	0.044	0.200**	0.044	0.155*	0.332**	0.448**	0.127	−0.230**	−0.188**

* *p < 0.05 and*

** *p < 0.01. CERQ-C, Chinese version of the Cognitive Emotion Regulation Questionnaire; MDD, major depressive disorder; SAI/TAI, State/Trait Anxiety Inventory; CES-D, Center for Epidemiologic Studies Depression Scale; CD-RISC, Connor-Davidson Resilience Scale*.

Table 4 presents the results of the final steps of the two hierarchical MRAs with regard to depression and anxiety with controlling for anxiety or depression. The first column shows the MRA for depression in healthy sample. Significant direct positive effects were found for catastrophizing, self-blame, rumination, and blaming others. Significant direct inverse effects were found for refocus on planning. The second column of Table 4 shows the MRA for anxiety in healthy sample, significant direct positive effects were showed for Catastrophizing, Positive reappraisal, and self-blame. Significant, inverse effects were found for acceptance and Refocus on planning. The third column shows the MRA for depression in adolescents with MDD with controlling for anxiety. The same significant direct positive effects were found for catastrophizing, rumination, blaming others, and acceptance. After controlling for depression, significant but low direct affect was found for Rumination, significant inverse effect was found for Refocus on planning.

**Table 4:** MRA on symptoms of depression and anxiety for high school students and adolescents with MDD.

**Strategy**	**High school students**				**Adolescents with MDD**			
	**Depression**		**Anxiety**		**Depression**		**Anxiety**	
	**β**	***t***	**β**	***t***	**β**	***t***	**β**	***t***
Self-blame	0.091	3.931**	0.051	2.095*	−0.168	2.534*	−0.081	−1.086
Acceptance	0.025	1.062	−0.092	−3.777**	0.147	2.174*	−0.003	−0.034
Rumination	0.089	3.763**	0.045	1.775	0.185	2.784**	0.149	2.002*
Positive refocus	0.014	0.599	−0.030	−1.217	−0.065	−0.935	−0.133	−1.742
Positive reappraisal	−0.020	−0.693	0.112	3.779**	−0.008	−0.102	0.100	1.149
Refocus on planning	−0.083	−2.856**	−0.111	−3.646**	−0.031	−0.384	−0.178	−2.011**
Putting into perspective	0.024	1.019	0.032	1.310	−0.031	−0.470	0.021	0.285
Catastrophizing	0.196	7.747**	0.108	3.983**	0.295	4.007**	0.159	1.885
Blaming others	0.063	2.779**	−0.027	−1.146	0.064	0.983	0.072	0.986
Model	*F*_(10, 1, 241)_ = 113.705; *p* < 0.001		*F*_(10, 1, 241)_ = 91.327; *p* < 0.001		*F*_(10, 189)_ = 13.296; *p* < 0.001		*F*_(10, 189)_ = 7.293; *p* < 0.001	
Explained variance (*R*^2^)	*R*^2^ = 0.48		*R*^2^ = 0.42		*R*^2^ = 0.43		*R*^2^ = 0.29	

* *p < 0.05 and*

** *p < 0.01. CERQ-C, Chinese version of the Cognitive Emotion Regulation Questionnaire; MDD, major depressive disorder; SAI/TAI, State/Trait Anxiety Inventory; CES-D, Center for Epidemiologic Studies Depression Scale; CD-RISC, Connor-Davidson Resilience Scale*.

### CFA

Indices obtained from structural equation modeling conducted to verify the nine-factor structure of the CERQ-C with Chinese respondents revealed that the modified nine-factor model generally fit the data of high school student group quite well, while the goodness of fit for the depression group was adequate to acceptable. Applying the nine-factor model to gender subgroups of high school student sample yielded acceptable model fits. The datasets from the initial test and the retest were each found to have an acceptable model fit. The index values obtained for each of the aforementioned groups or test times are reported in Table 5 with associated statistical values. These results indicate that the modified nine-factor model could serve as an initial model for subsequent measurement invariance tests.

**Table 5:** CFA of CERQ-C by group.

**Group**	**χ^**2**^**	***df***	**CFI**	**TLI**	**RMSEA (90%CI)**	**SRMA**
High school students (*N* = 1,253)	1993.566	558	0.923	0.932	0.045 (0.043–0.047)	0.048
Males (*N* =629)	1324.299	558	0.913	0.923	0.047 (0.043–0.050)	0.053
Females (*N* =624)	1340.153	558	0.924	0.933	0.047 (0.044–0.051)	0.051
Test, Time 1 (*N* =594)	1201.601	558	0.924	0.932	0.044 (0.041–0.048)	0.053
Retest, Time 2 (*N* =594)	1220.503	558	0.927	0.935	0.045 (0.041–0.048)	0.054
Adolescents with MDD (*N* =205)	927.064	558	0.901	0.912	0.057 (0.050–0.063)	0.063

*CFA, confirmatory factor analysis; CERQ-C, Chinese version of the Cognitive Emotion Regulation Questionnaire; MDD, major depressive disorder*.

### Measurement Invariance

Multiple-group CFA was used to examine measurement invariance of the CERQ-C across high school student and adolescents with MDD groups. Configural invariance was used to examine structural equivalence and whether the form or pattern of latent variables differed between the high school students and adolescents with MDD. All fit indices for configural equivalence were acceptable (Table 6) and thus configural equivalence was established. Then the configural model was adopted as a baseline model for subsequent analyses. Regarding metric equivalence, the factor loads of the high school student and adolescents with MDD groups were equal and other variables are estimated freely. All fit indices for metric equivalence were acceptable (Table 6), thereby establishing metric equivalence such that the model could be used as a baseline model for subsequent analyses. In scalar equivalence testing, conducted on the basis of the previous model with the intercept of the two groups set to be equal, all fit indices obtained were acceptable (Table 6), thereby establishing scalar equivalence. The fit index values obtained (Table 6) for strict equivalence were sufficient to establish strict invariance.

**Table 6:** Measurement invariance of the CERQ-C across presence of depression.

**Model**	**χ^**2**^**	***df***	**CFI**	**TLI**	**RMSEA (90%CI)**	**SRMA**	**Comparison**	**ΔCFI**	**ΔTLI**
Model 1	3394.610	1,116	0.925	0.915	0.052 (0.050–0.054)	0.050	–	–	–
Model 2	3017.959	1,143	0.927	0.920	0.047 (0.045–0.050)	0.052	2 vs. 1	0.002	−0.005
Model 3	3190.992	1,170	0.921	0.915	0.049 (0.047–0.051)	0.052	3 vs. 2	−0.006	−0.005
Model 4	3295.841	1,206	0.919	0.915	0.049 (0.047–0.051)	0.053	4 vs. 3	−0.002	0.000

*CERQ-C, Chinese version of the Cognitive Emotion Regulation Questionnaire; Model 1, configural invariance; Model 2, metric invariance; Model 3, scalar invariance; Model 4, strict invariance; χ^2^, chi-square goodness of fit; df , degrees of freedom; CFI, comparative fit index; TLI, Tucker-Lewis index; RMSEA, root mean square error of approximation; CI, confidence interval; Δ, difference*.

Index values obtained for configural invariance testing across gender groups (Table 7) confirmed structural equivalence of the CERQ-C across gender groups, thereby establishing configural equivalence. Using this model as the baseline, we assessed subsequent models. The successive fit indices obtained (Table 7) indicated that the metric, scalar, and strict invariance models were satisfactory, thus supporting the hypothesis that the CERQ-C has measurement invariance across gender. Finally, fit index values obtained for configural invariance testing across the test and 1-month retest time points, including indices for configural, metric, scalar, and strict invariance models (Table 8) also confirmed longitudinal measurement invariance.

**Table 7:** Measurement invariance of the CERQ-C across gender.

**Model**	**χ^**2**^**	***df***	**CFI**	**TLI**	**RMSEA (90%CI)**	**SRMA**	**Comparison**	**ΔCFI**	**ΔTLI**
Model 1	2664.207	1,116	0.919	0.928	0.047 (0.045–0.049)	0.052			
Model 2	2705.894	1,143	0.920	0.928	0.047 (0.044–0.049)	0.053	2 vs.1	0.001	0.000
Model 3	2795.892	1,170	0.919	0.925	0.047 (0.045–0.049)	0.053	3 vs. 2	0.001	0.003
Model 4	3006.659	1,206	0.913	0.917	0.049 (0.047–0.051)	0.054	4 vs. 3	0.006	0.008

*CERQ-C, Chinese version of the Cognitive Emotion Regulation Questionnaire; Model 1, configural invariance; Model 2, metric invariance; Model 3, scalar invariance; Model 4, strict invariance; χ^2^, chi-square goodness of fit; df, degrees of freedom; CFI, comparative fit index; TLI, Tucker-Lewis index; RMSEA, root mean square error of approximation; CI, confidence interval; Δ, difference*.

**Table 8:** Measurement invariance of the CERQ-C across time.

**Model**	**χ^**2**^**	***df***	**CFI**	**TLI**	**RMSEA (90%CI)**	**SRMA**	**Comparison**	**ΔCFI**	**ΔTLI**
Model 1	3767.437	2,297	0.935	0.928	0.033 (0.031–0.035)	0.051			
Model 2	3821.614	2,316	0.934	0.927	0.033 (0.031–0.035)	0.052	2 vs. 1	−0.001	−0.001
Model 3	3873.547	2,319	0.932	0.925	0.034 (0.032–0.036)	0.051	3 vs. 2	−0.002	−0.002
Model 4	3926.473	2,376	0.930	0.923	0.035 (0.033–0.037)	0.052	4 vs. 3	−0.002	−0.002

*CERQ-C, Chinese version of the Cognitive Emotion Regulation Questionnaire; Model 1, configural invariance; Model 2, metric invariance; Model 3, scalar invariance; Model 4, strict invariance; χ^2^, chi-square goodness of fit; df , degrees of freedom; CFI, comparative fit index; TLI, Tucker-Lewis index; RMSEA, root mean square error of approximation; CI, confidence interval; Δ, difference*.

### Comparisons of CERQ-C Scores Across Groups

Mean CERQ-C scale and subscale scores for compared groups are reported in Table 9 with their associated statistical values, including Cohen's d effect sizes. We found that, compared to high school students, the adolescents with MDD group tended to use the maladaptive ER strategies of Self-blame, Rumination, and Catastrophizing significantly more frequently, while tending to use the adaptive strategy of Refocus on planning less frequently. With respect to gender, girls had significantly higher Acceptance, Positive reappraisal, and Refocus on planning scores than boys, while boys had a significantly higher Catastrophizing score than girls.

**Table 9:** CERQ-C subscale mean score differences by presence of depression and gender.

**Strategy**	**Sample**		***t***	**Sig**.	**Cohen's d**	**Gender**		***t***	**Sig**.	**Cohen's d**
	**High school students**	**Adolescents with MDD**				**Boys**	**Girls**			
Self-blame	12.49 (2.356)	12.93 (2.445)	−2.455	0.014	0.183	12.38 (2.379)	12.61 (2.329)	−1.692	0.091	–
Acceptance	14.17 (2.870)	14.41 (2.828)	−1.118	0.264	–	13.98 (2.809)	14.37 (2.919)	−2.388	0.017	0.136
Rumination	12.52 (3.213)	13.21 (3.377)	−2.840	0.005	0.206	12.59 (3.189)	12.44 (3.238)	0.833	0.405	–
Positive refocus	12.67 (2.933)	12.49 (3.090)	0.815	0.415	–	12.61 (3.071)	12.74 (2.788)	−0.753	0.451	–
Positive reappraisal	14.38 (2.991)	14.28 (3.074)	0.458	0.647	–	14.01 (3.039)	14.75 (2.897)	−4.402	<0.01	0.249
Refocus on planning	13.98 (3.122)	13.49 (3.093)	2.089	0.037	0.157	13.67 (3.171)	14.30 (3.042)	−3.554	<0.01	0.202
Putting into perspective	10.45 (3.072)	10.80 (2.912)	−1.514	0.130	–	10.40 (3.158)	10.50 (2.984)	−0.577	0.564	–
Catastrophizing	8.18 (3.136)	10.19 (3.691)	−8.277	0.000	0.587	8.43 (3.242)	7.93 (3.007)	2.849	0.004	0.160
Blaming others	9.57 (2.701)	9.96 (2.837)	−1.930	0.054	–	9.69 (2.619)	9.44 (2.777)	1.619	0.106	–

*CERQ-C, Chinese version of the Cognitive Emotion Regulation Questionnaire; MDD, major depressive disorder*.

## Discussion

The purpose of this study was to provide information on the psychometric properties of the CERQ-C, which is a popular instrument for the evaluation of adolescents' use of cognitive ER strategies. Adequate reliability of the CERQ-C and its subscales was observed in high school student sample and we found better reliability in adolescents with MDD sample, with internal consistencies similar to those found for other versions of the CERQ [e.g., (6, 12)]. Our data affirmed that the CERQ-C can be considered to have adequate reliability in both high school students and adolescents with MDD groups.

The main precondition for the utility of a scale across groups is invariance of its factor structure (52). Here, our CFA supported the original nine-factor model for the CERQ-C in Chinese high school students and adolescents with MDD, consistent with previous studies carried out in other samples and other countries, including France (11), Spain (27), and a young adult sample in China (12). Therefore, the accumulated empirical evidence indicates that the nine-factor structure is retained across cultural contexts.

Recently, when McKinnon et al. (20) administrated the CERQ to 476 patients with MDD who were in remission, they found poor goodness of fit for a nine-factor structure but their analysis supported a five-factor structure instead. These latter findings raised an alert regarding the need to evaluate measurement equivalence of the CERQ between high school student and adolescents with MDD. The results of the present such examination support the configural, metric, and scalar invariance of the CERQ-C across the presence or absence of depression in Chinese adolescents, and thus indicate that group differences can be presumed to reflect true differences of latent variables. In our evaluations of measurement invariance of the CERQ-C across gender subgroups of our high school student sample and across time points, we obtained data supporting the instrument's configural, metric, scalar, and strict invariance across gender subgroups and over time. Thus, the present data indicate that meanings of CERQ-C concepts are maintained regardless of the presence of depression and are maintained across genders and over time.

But the results of our comparative analyses between high school student and adolescents with MDD groups are consistent with previous studies indicating that depressive adolescents are prone to using more maladaptive strategies than psychologically healthy people (53, 54). This finding supports the use of cognitive ER strategy training as an intervention for MDD.

Gender differences in the use of specific cognitive ER strategies have been reported, though the precise differences have been inconsistent. In our study, we found that female adolescents use Acceptance, Positive reappraisal, and Refocus on planning more often than male adolescents. In a previous study, Garnefski et al. (29) reported data suggesting that males use the strategy of Catastrophizing more than females, but the effect sizes of the differences were small. It is possible that gender differences in coping mechanisms used could reflect gender-specific cultural influences on ER strategies. Large inter-individual differences in ER strategies have been reported and attributed to different life experiences, events, and stressors (55). Thus, further exploration of measurement invariance of the CERQ across cultures is warranted.

Resilience plays an important role in adaption because it allows people to deal with stress. In the present analysis of the relationship between cognitive ER strategies and resilience among healthy high school students and adolescents with MDD, we all found that resilience correlated positively with a number of adaptive ER strategies (Acceptance, Positive refocus, Positive reappraisal, and Refocus on planning), consistent with previous studies [e.g., (56)]. Meanwhile, we found that resilience was inversely related to the maladaptive ER strategies of Catastrophizing and Blaming others. This pattern of findings suggests that teaching positive cognitive ER strategies, such as Positive reappraisal, to adolescents could buttress their resilience, enabling them to more readily extract positive meaning from adversity.

Coping strategies are closely associated with mental and psychological disorders. The risk and vulnerability of adolescents to stress and mental health problems increase with the application of maladaptive strategies (57). Indeed, our analyses showed that, as expected, cognitive strategies of Self-blame, rumination, and Catastrophizing would be the most important correlates of depression, while more use of Refocus on planning was associated with lower levels of anxiety and depression, consistent with findings in a prior sample of adolescents (58). Interestingly, we found positive reappraisal showed unique and specific positive relations to anxiety in healthy sample, differed from the previous studies (59, 61), further research may necessary to explore this strategy. The present study also found, for depression, significant direct positive effect for acceptance in adolescents with MDD, however, it is consistent with the previous studies in clinical samples (59, 60). Maybe for adolescents with MDD, Acceptance has identified as a passive form of resignation to negative experiences (62), so accepting poor outcomes might damage adolescents' self-efficacy, demoralized. Surely, whatever the directions of influence may be, it was clearly shown that certain cognitive ER strategies (especially maladaptive strategies) are related with depression and anxiety, and the result might suggest that “Refocus on planning” could be protective for adolescents, however, more researches was required to explored the role of cognitive ER strategies in the development of individual depressive and anxiety symptoms.

The limitations of this study should be considered when interpreting the results. First, because the present sample consisted of adolescents from only two high schools in Hunan Province and may not represent the general Chinese adolescent population. In the future, measurement equivalence of the CERQ-C should be reexamined in a nationally representative adolescent sample. Secondly, given that there were 205 participants in MDD group, especially only including 57 boys, and we didn't collect enough data in the retest neither. The sample size was too small to explore the gender equivalence and longitudinal equivalence of MDD group (63), if sample sizes are too small, indices used for model comparison in invariance testing are not always sensitive enough and inflate the Type I error rates (64). More MDD data could be collected to confirm the MI for CERQ in the future. Lastly, we did not follow-up to examine the developmental trend of cognitive ER strategies of healthy and depressive adolescents, which may be critical for directing the implementation of psychological interventions for depressed adolescents. There remains an urgent need for long-term tracking of coping efficacy in adolescents.

## Conclusion

In conclusion, the CERQ-C was demonstrated to be a valid and reliable instrument for the evaluation of cognitive ER strategies in Chinese adolescents. Measurement equivalence was established across presence of depression, gender, and time. These findings support the use of this revised instrument with the Chinese adolescent population, including adolescents with MDD.

## Data Availability Statement

The raw data supporting the conclusions of this article will be made available by the authors, without undue reservation.

## Ethics Statement

The studies involving human participants were reviewed and approved by Ethics Committee of Second Xiangya Hospital, Central South University. Written informed consent to participate in this study was provided by the participants' legal guardian/next of kin.

## Author Contributions

SY and DW designed and supervised the study including writing the paper. FD and XW performed the analysis and wrote paper and revised and approved the version to be published. FD, XW, CC, JH, and HZ collected the data and contributed to the analysis. All co-authors revised and approved the version to be published.

## Funding

This research was supported by the National Natural Science Foundation of China (Grant Number No. 82071532) given to SY.

## Conflict of Interest

The authors declare that the research was conducted in the absence of any commercial or financial relationships that could be construed as a potential conflict of interest.

## Publisher's Note

All claims expressed in this article are solely those of the authors and do not necessarily represent those of their affiliated organizations, or those of the publisher, the editors and the reviewers. Any product that may be evaluated in this article, or claim that may be made by its manufacturer, is not guaranteed or endorsed by the publisher.
